# Free Energy of Binding of Coiled-Coil Complexes with Different Electrostatic Environments: The Influence of Force Field Polarisation and Capping

**DOI:** 10.1007/s13659-014-0036-0

**Published:** 2014-08-22

**Authors:** Zhi-Li Zuo, Ling Guo, Ricardo L. Mancera

**Affiliations:** 1 State Key Laboratory of Phytochemistry and Plant Resources in West China, Kunming Institute of Botany, Chinese Academy of Sciences, Kunming, 650201 China; 2 College of Animal Husbandry & Veterinary, Liaoning Medical University, Jinzhou, 121001 China; 3 School of Biomedical Sciences, CHIRI Biosciences, Curtin University, GPO Box U1987, Perth, WA 6845 Australia

**Keywords:** Free energy of binding, c-Fos, c-Jun, Leucine zipper, Molecular dynamics, MM/GBSA, Coiled-coil

## Abstract

Coiled-coils are well known protein–protein interaction motifs, with the leucine zipper region of activator protein-1 (AP-1) consisting of the c-Jun and c-Fos proteins being a typical example. Molecular dynamics (MD) simulations using the MM/GBSA method have been used to predict the free energy of interaction of these proteins. The influence of force field polarisation and capping on the predicted free energy of binding of complexes with different electrostatic environments (net charge) were investigated. Although both force field polarisation and peptide capping are important for the prediction of the absolute free energy of binding, peptide capping has the largest influence on the predicted free energy of binding. Polarisable simulations appear better suited to determine structural properties of the complexes of these proteins while non-polarisable simulations seem to give better predictions of the associated free energies of binding.

## Introduction

Along with the linear interaction energy (LIE) [1, 2] and linear response approximation (LRA) [3, 4], the molecular mechanics/Poisson-Boltzmann surface area (MM/PBSA) and the molecular mechanics/generalised Born surface area (MM/GBSA) methods are popular rapid computational approaches used to predict the free energy of binding of biomolecules from molecular dynamics (MD) simulation trajectories. Polarisable force fields have also been used in the prediction of the free energy of binding using LRA [5] and MM/PBSA [6], but the improvement has been reported to be limited with respect to the increased computational cost.

The accurate calculation of the contributions of electrostatic interactions is very important in biophysical and biochemical modelling as many biological processes are regulated by electrostatic effects. Molecular charge distributions can be polarised when solvated in a high dielectric polarisable medium such as water. In traditional molecular mechanics force fields, such as AMBER [7], OPLS-AA [8], CHARMM [9], and GROMOS [10], electrostatic interactions are described using fixed charges independently of the environment. These non-polarisable force fields have had large success in molecular dynamics (MD) simulations and binding affinity predictions [11]. Polarisable force fields have been developed to allow changes in the charge distribution in response to the polarisable dielectric environment using induced point dipoles [12], classical Drude oscillators [13] and fluctuating charges [14, 15]. Polarisable force fields have been shown to be very promising and feasible for simulating the properties of ionic systems and small molecules [16]. Many studies have reported the validation of various newly developed polarisable force fields in simulations of large biomolecules (proteins, DNA/RNA, lipid bilayers and carbohydrates), showing improvements compared to additive force fields and in good agreement with experimental results [17–20].

The coiled coil protein motif consists of two or more α-helices wrapped around each other to form a left-handed supercoil, and is one of the most abundant protein interaction domains, involved in many biological processes. The leucine zipper domain, a typical coiled coil structure, is made up of the c-Fos–c-Jun heterodimer and is a part of the bZIP activator protein-1 (AP-1) transcription factor, which plays a crucial role in numerous cell pathways (proliferation, apoptosis, cell survival, differentiation) [21] and is often associated with numerous diseases, such as cancer and diabetes [22–24], making it an important therapeutic target. Various empirical approaches have been developed to predict the interactions of coiled-coil proteins [25]. Predictions of the free energy of binding of the c-Fos–c-Jun complex have been reported in an investigation of the influence of the choice of solvation model, protein force field and water potential [26]. It was found that the use of the AMBER polarisable force field ff02 in combination with the polarisable POL3 water potential results in a more stable secondary structure of this complex and a more consistent predicted free energy of binding with different simulation approaches (single-trajectory, multiple short-trajectory and independent-trajectory simulations). Furthermore, the predicted binding affinities of the series of c-Jun-based peptides targeting the c-Fos peptide also showed a good correlation with experimental melting temperatures, which provides the basis for the rational design of peptides based on internal, van der Waals, and electrostatic interactions [27].

Polarisation has been reported to have the opposite effect in dissimilar environments (i.e. in aqueous solutions vs. protein surfaces) in the calculation of free energies of binding of trypsin-benzamidine and trypsin-diazamidine complexes [28], which suggests that the effect of polarisation depends on the environment, especially in relation to electrostatic interactions. To explore the performance of polarisable protein force fields and water potentials in protein–protein interactions with different electrostatic environments, a comparison of long simulations using polarisable and non-polarisable force fields simulations of positively and negatively charged representative coiled-coil proteins was conducted in this work.

The optimised polarisable AMBER ff02.r1 force field/water POL3 potential combination and the non-polarisable AMBER ff99SB force field/water TIP3P potential combination were employed in MD simulations to characterise the effect of polarisation. The molecular mechanics generalised Born surface area (MM/GBSA) method was used following MD simulations to investigate the accuracy of predictions of the free energy of binding under different circumstances. The MM/GBSA method is a popular computational approach used to predict the free energy of interaction of biomolecules from MD simulation trajectories, giving efficient, reproducible and reliable calculations [29, 30], being also better for the prediction of the relative free energy of binding than MM/PBSA [31]. In particular, the use of different force fields and water potentials in combination with capping of the peptide termini in the c-Fos–c-Jun heterodimer and the c-Jun–c-Jun homodimer was investigated. These coiled coil protein complexes have opposite net charges: −4 for c-Fos–c-Jun and +2 for c-Jun–c-Jun.

## Materials and Methods

### Protein Structure Preparation

**Fig. 1:**
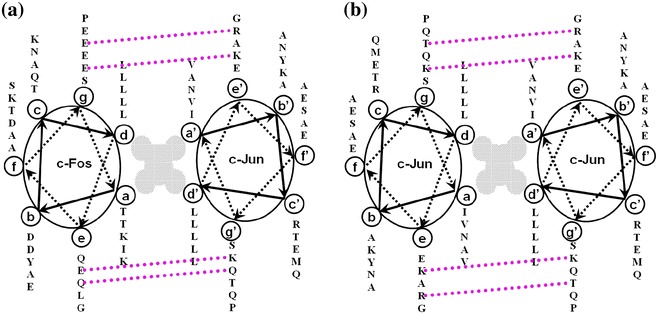
Helical wheel diagrams (looking down from the N-terminus to the C-terminus) for **a** c-Fos–c-Jun and **b** c-Jun–c-Jun complexes. Heptad residue positions are labelled *a* to *g* and *a′* to *g′*. Hydrophobic interactions are indicated by the *grey* region in the core of the complexes and potential ionic interactions are indicated by *dashed lines*

To make it possible to compare and validate our calculations with experiment, residues Ser 177 in c-Fos and Ser 301 in c-Jun were both mutated to tyrosine [32, 33], and the protein structure was constructed using the NMR structure of the c-Jun homodimer (PBD entry: 1JUN) and the crystal structure of the c-Fos–c-Jun complex (PBD entry: 1FOS). The amino acid sequence and the potential hydrophobic and hydrophilic interactions between the helices are shown in Fig. 1. The c-Fos peptide has the sequence ASTDTLQAETDQLEDEKYALQTEIANLLKEKEKLGAP, while the c-Jun peptide has the sequence ASIARLEEKVKTLKAQNYELASTANMLREQVAQLGAP. The free energies of binding of the c-Fos–c-Jun heterodimer and c-Jun–c-Jun homodimer have been determined experimentally to be −4.1 and −4.8 kcal/mol, respectively [32, 33]. Uncharged acetylated (ACE/NME) termini are often used to cap the truncated peptide bonds at the terminal ends of a protein or peptide to help to stabilise the structure and prevent helical ends from fraying in simulations of short alpha helices [34]. In this work, terminal residues were capped with an ACE (acetyl beginning group) and NME (N-methylamine ending group) in the N-terminus and C-terminus, respectively. All Lys, Arg, Glu and Asp residues were kept in their ionised forms during the simulations by assuming a neutral pH.

### MD Simulations

The AMBER 10.0 program [35, 36] with the non-polarisable ff99SB [35] and polarisable ff02.r1 [37, 38] force fields were used in the energy minimisations and MD simulations. The protein complexes were solvated in cubic boxes of TIP3P [39] and POL3 [40] water molecules, in combination with the non-polarisable force field and polarisable force fields, respectively. Approximately 5600–5900 water molecules were added with a minimum distance of 12.0 Å between each face of the box and the protein complex. Four Na^+^ and 2 Cl^−^ counterions were added to neutralise the net charge of the proteins to the c-Fos-c-Jun and c-Jun-c-Jun simulation systems, respectively. In all simulations, long range electrostatic interactions were calculated using the particle mesh Ewald (PME) method [41] with a 1.0 Å grid spacing and a fourth-order spline for interpolation. All bonds involving hydrogen atoms were constrained using the SHAKE algorithm [42] and the non-bonded cut-off was set to 10.0 Å. All simulations were carried out in the isobaric-isothermal (NPT) ensemble with an external isotropic pressure of 1 atm using weak coupling to a pressure bath [43] and a temperature of 293 K (which is the reference temperature of circular dichroism determinations) using the Berendsen thermostat algorithm with a 2 ps^−1^ collision frequency. The simulations were carried out with a time step of 1.0 fs. Periodic boundary conditions were applied throughout.

Unfavourable steric contacts in the initial configuration of each simulation system were removed by energy minimisation. A stepwise restraint releasing strategy was used. Initially 5000 steps of steepest descents and 5000 steps of conjugate gradients energy minimisation were carried out in which a restraining force (10.0 kcal mol^−1^ Å^−2^) was applied to all protein atoms. After this a further combination of 5000 steps of steepest descents and 5000 steps of conjugate gradients energy minimisation were carried out in which the same restraining force was applied to all backbone atoms only. Finally 10,000 steps of energy minimisation were carried out without any restraining force. The systems were then heated from 0 to 293 K over 50 ps under constant volume and temperature conditions (NVT). The stepwise restraint releasing strategy was used again to equilibrate the systems under NPT conditions at 1 atm and 293 K: a weak restraining force (5.0 kcal mol^−1^ Å^−2^) was applied to the whole protein over 50.0 ps, followed by a further 50.0 ps with the same restraining force applied only on all backbone atoms, and then finally 25.0 ps without any restraints. The production phases of the simulations were then run without any restraints at 293 K for 100 ns for each protein system. Various properties (density, temperature, pressure, kinetic and potential energies) were monitored during the simulations and configuration snapshots were saved every 10 ps.

To monitor the conformational changes during the simulations, the root mean squared deviation (RMSD) between the initial coordinates of the backbone atoms of the proteins and the coordinates along the MD simulations was computed for every snapshot. The total α-helicity of the proteins was calculated employing the DSSP (Defined Secondary Structure of Proteins) definitions, whereby helicity was taken as the number of helical residues determined by DSSP [44]. Helical propensity was taken as the percentage helicity of all amino acid residues.

### Free Energy Calculations

The free energy perturbation (FEP) formalism shows that the free energy can be decomposed into various terms (electrostatic, non-polar or hydrophobic, and entropic contributions) and/or different groups of atoms. The MM/GBSA method was used to estimate the free energies of binding between the peptides in the c-Fos–c-Jun and c-Jun–c-Jun complexes from the snapshots collected from the MD simulations.

The MM/GBSA method typically involves the calculation of the molecular mechanics gas-phase energies, continuum electrostatic solvation energies (by solving the generalised Born equation), surface area-based non-polar energies, and various entropic terms [45, 46].

1 $$G=E_{MM}+G_{GBSA}\;-\;TS$$

where *E*_*MM*_ is the average molecular mechanics energy, *G*_*GBSA*_ is the free energy of solvation, and *TS* is the entropy. The molecular mechanics energy is the sum of the average van der Waals energy (*E*_*vdw*_), the average electrostatic energy (*E*_*elec*_), and the average internal energy (*E*_*int*_), which includes internal bond stretching, bond bending and torsional angle energies. The *TS* term is the sum of translational, rotational and vibrational entropies. The translational and rotational entropies are approximated by statistical mechanics equations of molecules in the gas phase [45, 47], while vibrational entropies can be approximated through normal modes analysis [48].

*G*_*GBSA*_ is the free energy of solvation, given by

2 $$G_{GBSA}=G_{GB}+G_{SA}$$

where *G*_*GB*_ is the electrostatic component of the free energy of solvation, calculated by solving the generalised Born equation [49]. *G*_*SA*_ is the non-polar contribution to the free energy of solvation, which is calculated from the solvent-accessible surface area (SASA) [50]. This term is computed with the equation *G*_*SA*_ = *γSA*, where *SA* is the solvent-accessible surface area (calculated, for example, by the MSMS program [51]) and *γ* is a parameterised constant (*γ* = 0.0072 kcal mol^−1^ Å^−2^).

The free energy (Δ*G*) of the binding of two peptides can be thus calculated as:

3 $$DG\;=\;G_{complex}-\;G_{peptide1}-\;G_{peptide2}$$

MM/GBSA calculations were carried out using the *PBSA* module within the AMBER 10.0 program.

## Results and Discussion

The effects of polarisation, the addition of terminal residue caps and the net charge of the coiled-coil peptides on the calculation of the free energy of binding were investigated.

## Stability Comparison

**Fig. 2:**
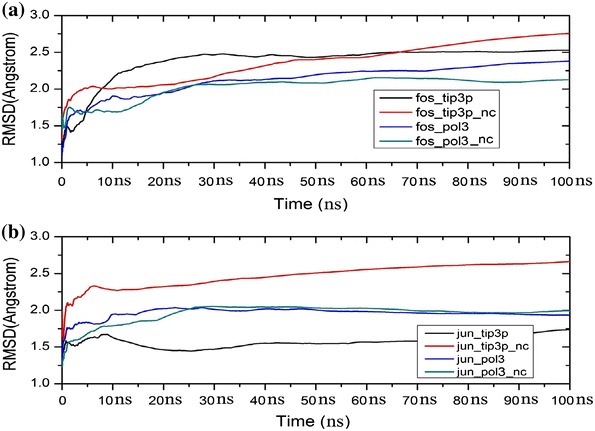
Plots of the running average of the RMSD calculated for all backbone atoms during MD simulations with and without capping (nc) using the ff99SB/TIP3P and ff02.r1/POL3 force field and water potential combinations: **a** c-Fos–c-Jun complex; **b** c-Jun–c-Jun complex

The stability of the c-Fos–c-Jun and c-Jun–c-Jun coiled-coil complexes with and without capping was first assessed by the calculation of the running average of the RMSD of all backbone atoms, as shown in Fig. 2. For the c-Fos–c-Jun complex with capping, the RMSD at the end of 100 ns of simulation was found to be 2.4 Å (ff02.r1/POL3) and 2.5 Å (ff99SB/TIP3P), while it was found to be 2.2 Å (ff02.r1/POL3) and 3.2 Å (ff99SB/TIP3P) without caps. In the case of the c-Jun–c-Jun complex with capping, the final average RMSD was found to be 1.9 Å (ff02.r1/POL3) and 1.7 Å (ff99SB/TIP3P) while it was found to be 2.0 Å (ff02.r1/POL3) and 2.7 Å (ff99SB/TIP3P) without capping. As in our previous study [26], the overall RMSD values calculated in simulations using the polarisable ff02.r1/POL3 combination are generally smaller than when using the non-polarisable ff99SB/TIP3P combination. It appears that there are no significant differences between the RMSD of the c-Jun–c-Jun complex with and without capping with the polarisable ff02.r1/POL3 combination, but a significant increase in RMSD can be seen when using the non-polarisable ff99SB/TIP3P combination with no capping. A similar behaviour is observed with the c-Fos–c-Jun complex, where the use of the non-polarisable ff99SB/TIP3P with no capping leads to a steady increase in the RMSD, even after 100 ns simulation. Capping was initially added to make the structure stable during MD simulation. However, it is unexpected that the use of the polarisable ff02.r1/POL3 combination with the c-Fos–c-Jun complex results in an increase in RMSD when capping is used. According to the RMSD values, simulations using the non-polarisable ff99SB/TIP3P combination reveal that the c-Jun–c-Jun homodimer is more stable than the c-Fos–c-Jun heterodimer, which is consistent with the higher stability of the former compared to the latter, since the free energy of binding of the c-Fos–c-Jun complex is −4.1 kcal/mol and that of the c-Jun–c-Jun homodimer is −4.8 kcal/mol [32, 33]. It can be concluded that the use of the polarisable ff02.r1/POL3 combination may not require the use of capping to improve stability. At the same time, the use of the non-polarisable ff99SB/TIP3P combination appears to require the use of capping to prevent a significant loss in stability.

**Fig. 3:**
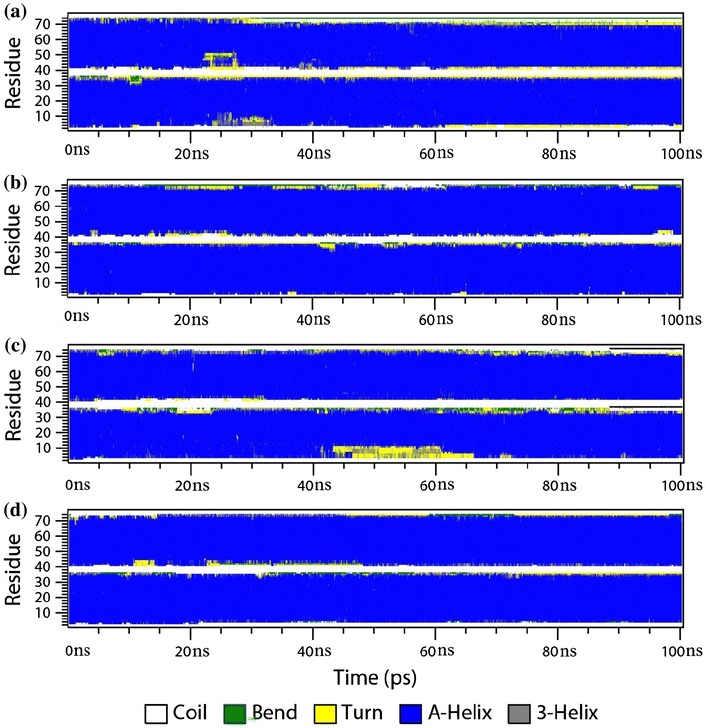
Time evolution of the secondary structure profile of c-Fos–c-Jun complex: **a** no capping using the non-polarisable ff99SB/TIP3P combination; **b** no capping using the polarisable ff02.r1/POL3 combination; **c** with capping using the non-polarisable ff99SB/TIP3P combination; and **d** with capping using the polarisable ff02.r1/POL3 combination

**Fig. 4:**
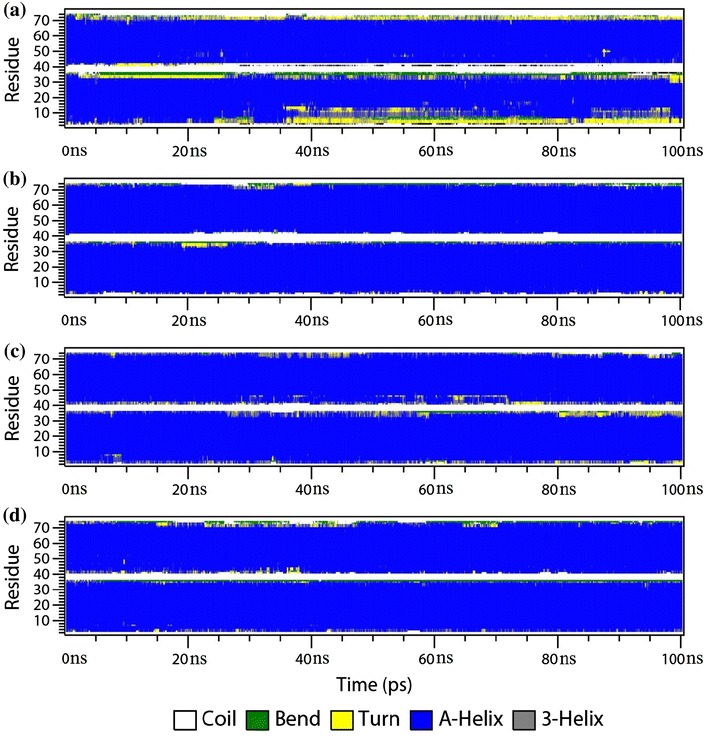
Time evolution of the secondary structure profile of c-Jun–c-Jun complex: **a** no caps using the non-polarisable ff99SB/TIP3P combination; **b** no caps using the polarisable ff02.r1/POL3 combination; **c** with caps using the non-polarisable ff99SB/TIP3P combination; and **d** with caps using the polarisable ff02.r1/POL3 combination

The overall stability of both complexes was also assessed using DSSP analysis. Figures 3 and 4 illustrate the time evolution of the secondary structure profiles of the c-Fos–c-Jun and c-Jun–c-Jun complexes, respectively, comparing the use of polarisable and non-polarisable force fields with and without terminal capping. The DSSP secondary structure profiles are correlated very well with RMSD analysis. It can be seen that most of changes in secondary structure occur at the termini of the peptides, whether it is the c-Fos peptide or the c-Jun peptide. And the secondary structural change is getting bigger along the time of simulation as indicated more and more yellow areas from DSSP profile at the first 20 ns.

Use of the polarisable ff02.r1/POL3 combination appears to result in a slightly higher α-helical content (helical propensity) in all structures: when capping is used, 85.7 % for the c-Fos–c-Jun heterodimer and 86.6 % for the c-Jun–c-Jun homodimer, whereas when no capping is used the helical content is again 85.7 % for the c-Fos–c-Jun heterodimer and slightly lower with 85.9 % for the c-Jun–c-Jun homodimer. On the other hand, when the non-polarisable ff99SB/TIP3P combination is used, the α-helical content is on average lower: when capping is used, 80.7 % for the c-Fos–c-Jun heterodimer and 86.2 % for the c-Jun–c-Jun homodimer, whereas when no capping is used the helical content is lower for both the c-Fos–c-Jun heterodimer (79.6 %) and the c-Jun–c-Jun homodimer (73.4 %). These findings are consistent with the measured RMSD values, revealing that the use of capping results in more stable structures that exhibit less changes to their secondary structures, but this effect is more consistently observed with the use of the non-polarisable ff99SB/TIP3P combination. The use of the ff02.r1/POL3 polarisable combination, on the other hand, results in more stable structures with higher helical propensities. These findings are also in agreement with earlier reports indicating that protein polarisation is important in reducing overall structure fluctuations and making the protein structure more rigid [52, 53], due to the fact that electronic polarisation is critical for stabilising hydrogen bonding, which is the dominant interaction in protein secondary structures [54].

The charges at the peptide termini are known to affect helix stability, with the neutral blocking NME and ACE groups of the termini stabilising the protein structure [55]. This is consistent with the present simulations using the ff99SB/TIP3P combination with capping, which show increased stability as measured by overall RMSD and secondary structure content. The higher RMSD values measured in the simulations using the polarisable force field combination with capping may arise from the fact that the partial charges of the ACE and NME groups in the ff02 force field may affect backbone conformation [17]. In the ff02.r1 force field used in this study, the atomic charges of the backbone torsion parameters were re-optimised with ACE-Ala-NME and ACE-Ala_7_-NME, which might be inappropriate for the peptides in our simulations (ACE-X-NME, X = 37) as they are much longer. It is thus unclear whether this charge parameterisation may be responsible for the higher stability of the peptide complexes investigated here without capping in the polarisable simulations.

## Free Energies of Binding

To measure the strength of interaction between the two peptides in the c-Fos–c-Jun and c-Jun–c-Jun complexes, the absolute free energy of binding was calculated using the MM/GBSA method. Tables 1 and 2 list the free energies of binding (and its various energy terms) of these peptides with and without capping using both the polarisable ff02.r1/POL3 and non-polarisable ff99SB/TIP3 combinations. There are three structures of the c-Jun peptide that can be taken from the NMR and X-ray structures considered here (c-Jun1, c-Jun2, and c-Jun), and the predicted values of the absolute free energy (*G*_*mmgbsa*_) are relatively consistent. Use of the polarisable ff02.r1 force field and POL3 water potential combination resulted consistently in lower absolute free energies of binding compared to the use of the non-polarisable ff99SB force field and TIP3P water potential combination, which is consistent with our earlier reports of lower free energies of binding calculated using polarisable force fields [27].

**Table 1:** The free energy of binding components of the c-Fos–c-Jun complex with/without capping using the ff98SB/TIP3P and ff02.r1/POL3 force field and water potential combinations (kcal/mol)

	*E*_*ele*_	*E*_vdw_	*E*_int_	*G*_gbsa_	*G*_*mmgbsa*_	*TS*	*G*_bind-gb_
POL3							
c-Fos	−692.8/−755.7	−72.8/−73.2	792.3/775.0	−1198.1/−1219.3	−1171.4/−1273.3	481.7/470.9	−1653.1/−1774.2
c-Jun	−1314.1/−1208.4	−67.0/−68.1	806.2/786.8	−600.4/−787.5	−1175.3/−1277.2	475.6/468.3	−1650.9/−1745.5
Cplx	−2377.6/−2272.6	−236.9/−231.0	1598.5/1561.8	−1397.0/−1664.7	−2413.0/−2606.5	904.5/887.7	−3317.5/−3494.2
Delta	−370.8/−308.4	−97.0/−89.7	0.0/0.0	401.6/342.1	−66.2/−56.0	−52.7/−51.5	−13.5/−4.5
TIP3P							
c-Fos	−526.2/−535.0	−79.3/−75.5	785.1/763.8	−1181.7/−1268.3	−1002.1/−1115.0	480.9/474.4	−1483.0/−1588.6
c-Jun	−1143.3/−1093.0	−76.8/−61.6	796.4/775.5	−612.4/−756.9	−1036.0/−1135.9	477.3/466.4	−1513.4/−1602.7
Cplx	−2008.1/−1878.4	−247.2/−213.9	1581.5/1539.3	−1429.0/−1742.5	−2102.7/−2295.5	903.3/892.5	−3006.1/−3189.5
Delta	−338.6/−250.4	−91.1/−76.9	0.0/0.0	365.1/282.7	−64.6/−44.6	−55.0/−48.3	−9.7/+3.7

**Table 2:** The free energy of binding components of the c-Jun–c-Jun complex with/without capping using the ff98SB/TIP3P and ff02.r1/POL3 force field and water potential combinations (kcal/mol). One of the peptides in the complex is labeled as c-Jun1 and the other one c-Jun2

	*E*_*ele*_	*E*_vdw_	*E*_int_	*G*_gbsa_	*G*_*mmgbsa*_	*TS*	*G*_bind-gb_
POL3							
c-Jun1	−1326.5/−1197.8	−68.6/−68.2	803.5/785.3	−586.3/−797.9	−1177.9/−1278.6	475.8/467.8	−1653.6/−1746.4
c-Jun2	−1342.7/−1185.4	−69.8/−68.4	805.5/787.1	−567.8/−807.4	−1174.8/−1274.1	473.3/468.5	−1648.1/1742.6
Cplx	−2620.9/−2225.6	−230.0/−229.2	1609.0/1572.4	−1168.8/−1721.9	−2410.7/−2604.2	901.6/888.6	−3312.3/−3492.8
Delta	48.3/157.6	−91.6/−92.5	0.0/0.0	−14.7/−116.6	−58.1/−51.5	−47.5/−47.8	−10.6/−3.7
TIP3P							
c-Jun1	−1251.8/−1042.8	−78.0/−72.3	817.1/782.8	−521.0/−739.4	−1033.8/−1071.7	473.0/473.4	−1506.7/−1545.1
c-Jun2	−1299.5/−1180.4	−77.7/−69.3	819.4/788.6	−480.6/−643.5	−1038.4/−1104.6	470.3/468.9	−1508.7/−1573.5
Cplx	−2503.5/−2143.9	−231.4/−224.7	1636.5/1571.4	−1026.1/−1429.7	−2124.5/−2226.9	897.8/894.1	−3022.3/−3121.0
Delta	47.8/79.2	−75.7/−83.1	0.0/0.0	−24.5/−46.7	−52.3/−50.6	−45.4/−48.2	−6.9/−2.4

**Fig. 5:**
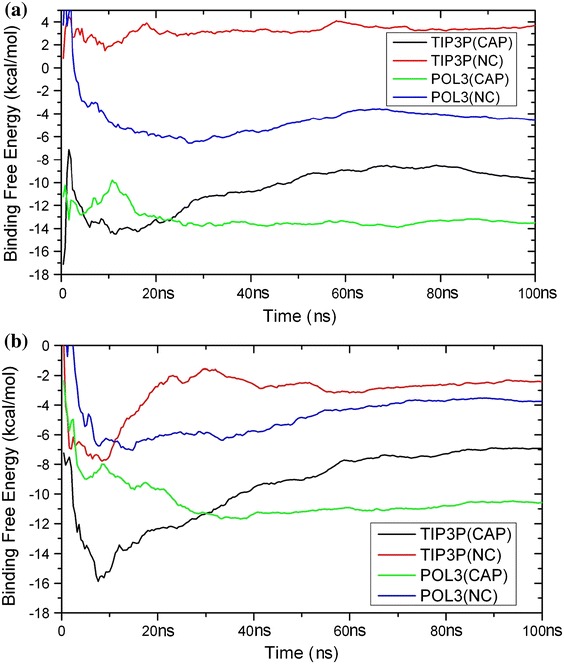
Running average of the free energy of binding with capping (CAP) and without capping (NC) using the non-polarisable ff99SB/TIP3P combination and the polarisable ff02.r1/POL3 combination: **a** c-Fos–c-Jun complex; **b** c-Jun-c–Jun complex

To investigate the convergence of the free energy of binding as a function of simulation time, the running averages of the free energy of binding for each complex are shown in Fig. 5. The running averages were calculated both with and without caps using both the polarisable and non-polarisable force field combinations. It can be seen that in most simulations the free energies of binding converge within 20 ns, as the running averages become reasonably stable. The only exception is the non-polarisable simulation of c-Jun–c-Jun without caps, which converged after 40 ns.

The predicted free energies of binding do not match experimental determinations very well: −4.1 kcal/mol for the c-Fos–c-Jun heterodimer and −4.8 kcal/mol for the c-Jun–c-Jun homodimer. Nonetheless, predictions of the absolute free energy of binding using the polarisable ff02.r1/POL3 combination without capping are closer to experimental values for both complexes, with predicted values of −4.5 kcal/mol for c-Fos–c-Jun and −3.7 kcal/mol for c-Jun–c-Jun. These values are of similar magnitude to experimental ones but do not predict correctly that the binding of the c-Jun–c-Jun homodimer is stronger.

It is more reasonable to study the relative free energy of binding between each complex, as the empirical terms would be cancelled for these empirical approaches to calculated free energy of binding, such MM/GBSA. Interestingly, capping seems to have a larger effect on the free energy of binding than from the use of a polarisable over a non-polarisable force field and water potential combination. This is revealed by the relative free energy of binding for both complexes: *G*_bind-gb_(ff02.r1/POL3 with capping) < *G*_bind-gb_(ff99SB/TIP3P with capping) < *G*_bind-gb_(ff02.r1/POL3 without capping) < *G*_bind-gb_(ff99SB/TIP3P without capping). The two intermediate options, i.e., simulations using either the ff02.r1/POL3 or the ff99SB/TIP3P combination without capping, result in similar accuracy compared with experiment.

As the calculation of GB solvation energies was carried out with the standard *PBSA* module in AMBER 10.0, it did not include the effect of polarisation directly as the solvent is treated as a polarisable dielectric continuum [6]. Since the MM/GBSA method is used to analyse snapshots from an MD simulation, the polarisation energy as computed by a polarisable force field is not replicated. The effect of polarisation is thus an indirect one arising from differences in the structures generated during simulations using polarisable force fields. Consequently, the overestimation of the magnitude of electrostatic interactions computed by the MM/GBSA method (see below) is unlikely to be improved upon analysis of trajectories from simulations using polarisable force fields [56, 57]. This might be the reason why the free energies of binding predicted with the polarisable simulations show bad agreement with experimental data.

As discussed above, the coiled-coil complexes in the polarisable simulations were determined to be more stable with capping than without it. The predicted free energies of binding show a similar trend: *G*_bind-gb_(ff02.r1/POL3 with capping) is lower than that without capping. As can be seen in Tables 1 and 2, the ∆*E*_ele_ of binding of the complexes with capping in the polarisable simulations is more negative compared to the results obtained with the non-polarisable simulations. The ∆*E*_ele_ of the complexes without capping is larger (it increases from 48.3 to 157.6 kcal/mol for the c-Jun–c-Jun complex) in the polarisable simulations. These distinct differences in the electrostatic contribution to the free energy of binding may be the reason why predictions of the free energy of binding for complexes without capping are relatively more accurate.

## Electrostatic Interactions and Solvation Energies

The energy terms collected in Tables 1 and 2 reveal that the free energies are dominated by the electrostatics and solvation terms, with the free energies of solvation (∆*G*_*GBSA*_) and the electrostatic energies (∆*E*_ele_) having opposing values between the c-Fos–c-Jun heterodimer and the c-Jun–c-Jun homodimer in simulations using either the polarisable ff02.r1/POL3 or the non-polarisable ff99SB/TIP3P force field combination. The ∆*G*_*GBSA*_ is positive and the ∆*E*_ele_ is negative in the c-Fos–c-Jun complex, while the ∆*G*_*GBSA*_ is negative and the ∆*E*_ele_ is positive in the c-Jun–c-Jun complex. It can be seen that the electrostatic energy term is favourable for binding whereas the solvation term is unfavourable in the c-Fos–c-Jun complex, while the opposite situation arises in the c-Jun–c-Jun complex. This is likely to result from the fact that the peptides have net opposite charges: if only charged residues at the interface between the peptides are considered (at positions *e* or *g*), the c-Fos peptide has a net charge of −4 while the c-Jun peptide has a net charge of +3. More importantly, most of the residue pairs in the potential electrostatic interactions, as indicated in Fig. 1, are oppositely charged in the c-Fos–c-Jun complex, while they have the similar charges in the c-Jun–c-Jun complex. From the point of view of electrostatic interactions only, the presence of these charges suggests that the c-Fos and c-Jun peptides will bind each other favourably, whereas the homodimerisation of c-Jun will result in a net repulsion. The relative high electrostatic energy (−692.8 and −526.2 kcal/mol for the polarisable and non-polarisable calculations, respectively) for the c-Fos peptide indicate that it is less stable in the gas phase, compared to the c-Jun peptide, which has a more negative energy by −620 kcal/mol. The presence of opposite charges in its partner peptide, c-Jun, results in stronger electrostatic interactions between the two peptides, producing a more negative ∆*E*_ele_. Furthermore, as a consequence of the different charges in each peptide, the counteracting solvation energies will be substantially different. The c-Fos peptide with a larger net charge gives rise to a positive ∆*G*_*GBSA*_ upon complexation with c-Jun. Use of a polarisable force field magnifies this effect by providing a more negative ∆E_ele_ for the formation of the c-Fos–c-Jun complex and a more positive ∆E_ele_ for the formation of the c-Jun–c-Jun complex. The *E*_ele_ for both of c-Fos and c-Jun is more negative when the polarisable ff02.r1/POL3 combination is used.

## Conclusions

We have investigated the effect of polarisation with and without peptide terminus capping on the prediction of protein stability and free energy of binding of the coiled-coil protein complexes c-Fos–c-Jun and c-Jun–c-Jun complexes. The AMBER polarisable (ff02.r1) and non-polarisable (ff99SB) force fields were considered in combination, respectively, with the polarisable POL3 and non-polarisable TIP3P water potentials.

Both polarisation and terminal capping have been found to increase the stability of the c-Fos–c-Jun and c-Jun–c-Jun coiled-coil complexes. This is revealed by lower RMSD values in simulations using the ff02.r1/POL3 polarisable force field combination compared to the ff99SB/TIP3P one. The use of terminal capping appears to have significantly reduced the RMSD values in the simulation using the non-polarisable ff99SB/TIP3P combination; however, it may not be required to improve the stability using the ff02.r1/POL3 combination. Secondary structure analyses further confirmed these findings as revealed by higher helical propensities when capping is used.

NME and ACE terminal groups were used to block terminal residues, neutralising the charges and stabilising the structure of the peptide complexes in the simulations using the non-polarisable ff99SB/TIP3P combination. In the case of simulations using the polarisable ff02.r1/POL3 combination, it is possible that the formation of hydrogen bonds by the terminal residues with other residues and the solvent may be reduced, as the electronic polarisation in hydrogen bonds is critical for the stability of proteins. The parameterisation of charges for the ACE and NME groups was originally carried out using ACE-Ala-NME and ACE-Ala_7_-NME peptides, which are much shorter than the peptides studied in this work, which may have an indirect effect on torsional parameters. These observations might be helpful for the further development of protein polarisable force fields.

The change of molecular mechanics energy (*E*_MM_) and the free energy of solvation (*G*_GBSA_) can be influenced significantly by the electrostatic properties of the peptides. The interaction of peptides with opposite charges can result in a negative *E*_MM_ and a positive *G*_GBSA_, whereas the interaction of peptides with the same charges can result in a positive *E*_MM_ and a negative *G*_GBSA_. The electrostatic properties of coiled-coil peptides are thus important in their interactions, with oppositely charged residues required to achieve stronger binding.

The main finding of this work is that capping of the peptide termini appears to have a larger effect on the absolute free energy of binding than using a polarisable over a non-polarisable force field and water potential combination. When comparing polarisable and non-polarisable simulations, it would appear that polarisable simulations could be used to determine structural properties while non-polarisable simulations are better suited for the prediction of free energies of binding. Two simulation protocols, i.e., simulations using ff02.r1/POL3 without capping and ff99SB/TIP3P without capping can be used for any further work to explore coiled-coil interaction energies.
